# Plasticity of Membrane Binding by the Central Region of α-Synuclein

**DOI:** 10.3389/fmolb.2022.857217

**Published:** 2022-06-15

**Authors:** Carlos Navarro-Paya, Maximo Sanz-Hernandez, Alfonso De Simone

**Affiliations:** ^1^ Department of Life Sciences, Imperial College London, London, United Kingdom; ^2^ Department of Pharmacy, University of Naples Federico II, Naples, Italy

**Keywords:** α-synuclein, intrinsically disordered proteins, vesicle clustering, double-anchor mechanism, membrane binding, coarse-grained simulations

## Abstract

Membrane binding by α-synuclein (αS), an intrinsically disordered protein whose aggregation is associated with Parkinson’s disease, is a key step in determining its biological properties under both physiological and pathological conditions. Upon membrane interaction, αS retains a partial level of structural disorder despite acquiring α-helical content. In the membrane-bound state, the equilibrium between the helical-bound and disordered-detached states of the central region of αS (residues 65–97) has been involved in a double-anchor mechanism that promotes the clustering of synaptic vesicles. Herein, we investigated the underlying molecular bases of this equilibrium using enhanced coarse-grained molecular dynamics simulations. The results enabled clarifying the conformational dependencies of the membrane affinity by this protein region that, in addition to playing a role in physiological membrane binding, has key relevance for the aggregation of αS and the mechanisms of the toxicity of the resulting assemblies.

## Introduction

α-Synuclein (αS) is an intrinsically disordered protein whose aggregation is linked to neurodegenerative diseases collectively known as synucleinopathies (Uversky and Eliezer, 2009; Lashuel et al., 2013), including Parkinson’s disease (PD) (Dobson, 2003; Bosco et al., 2006; Luk et al., 2012; Goedert et al., 2013; Jucker and Walker, 2013; Ikenoue et al., 2014; Luth et al., 2014; Paslawski et al., 2014; Chiti and Dobson, 2017; Ahmed et al., 2020), dementia with Lewy bodies (Galvin et al., 1999), and multiple system atrophy (Spillantini et al., 1998). Aggregates of αS are major constituents of intracellular deposits—Lewy bodies—in PD, whereas mutations, duplications, and triplications of the αS-encoding gene (SNCA) have been associated with early-onset forms of this disease (Polymeropoulos et al., 1997; Singleton et al., 2003). While the pathological relevance of αS is generally established, its function remains elusive, although growing evidence points to a role in synaptic vesicles (SVs) trafficking (Auluck et al., 2010; Burre, 2015). A recursive feature in most of the putative functions of αS involves binding to biological membranes (Lorenzen et al., 2014; Snead and Eliezer, 2014; Fusco et al., 2018; Jacob et al., 2021), an interaction relevant to the normal form of αS *in vivo* (Newberry et al., 2020a; Newberry et al., 2020b) and influencing its aggregation (Perrin et al., 2001; Zhu and Fink, 2003; Breydo et al., 2012; Comellas et al., 2012; Galvagnion et al., 2015; Antonschmidt et al., 2021) and the toxicity of its oligomeric aggregates (Fusco et al., 2017). This interaction has been observed in different biological contexts, including the regulation of the homeostasis of SVs during neurotransmitter release (Wislet-Gendebien et al., 2006; Auluck et al., 2010), the localization to mitochondrial membranes or mitochondrial-associated membranes (Maltsev et al., 2013; Plotegher et al., 2014; Menges et al., 2017; Ordonez et al., 2018; Ramezani et al., 2019), where it has been proposed to mitigate the effects of oxidative stress, or the binding to lysosomal membranes (Bourdenx et al., 2014).

Upon membrane binding, αS undergoes a folding transition by adopting α-helical conformation throughout the N-terminal 90 residues of its sequence (Bodner et al., 2009; Maltsev et al., 2012). This ordering process is promoted by imperfect sequence repeats of 11-residues that can fold into amphipathic α-helices (Eliezer et al., 2001) that bind lipid membranes by laying parallel at the interface between the polar lipid heads and the hydrophobic interior of the membrane (Cheng et al., 2013; Fusco et al., 2014; Fusco et al., 2016a). The modular organization of seven imperfect repeats in the αS sequence provides αS with the ability to adapt its membrane binding to a large variety of amphipathic assemblies, ranging from small detergent micelles to lipid vesicles and membranes (Ulmer et al., 2005; Jao et al., 2008; Bodner et al., 2009) as well as to the water–air interface (Campioni et al., 2014).

**FIGURE 1:**
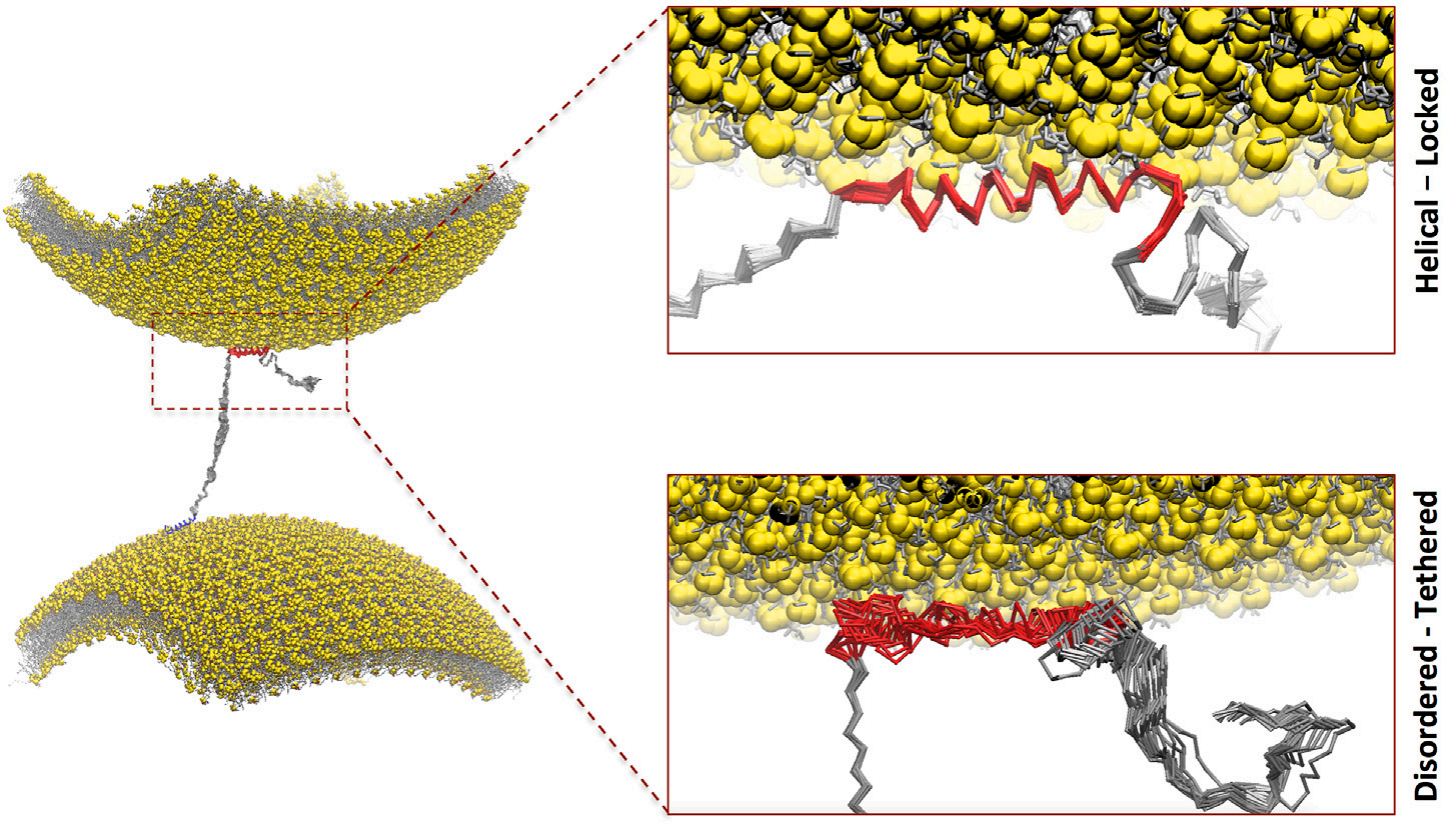
Double-anchor mechanism by which one molecule of αS binds across two vesicles. In particular, αS interacts with a first vesicle (lower in the plot) *via* the N-terminal anchor adopting an amphipathic α-helical conformation (blue) and a second vesicle (upper in the plot) *via* the region 65–97 (red). The present study focused on the conformational dependencies of membrane interaction in the region 65–97 by studying the binding *via* helical-locked (top insert) and disordered-tethered (bottom insert) conformations.

NMR studies with small unilamellar vesicles (SUVs) mimicking the lipid composition of SVs identified three major αS regions with distinct structural and dynamical properties in their membrane-bound state (Fusco et al., 2014). These include an N-terminal α-helical segment, effectively anchoring the protein on the membrane by partial insertion of the N-terminal 12 residues (Fusco et al., 2016a), an unstructured C-terminal region that weakly associates with the membrane, and a central region that undergoes order–disorder transitions at the membrane surface and that determines the overall affinity for lipid bilayers (Fusco et al., 2014). It was observed that the membrane interactions of the N-terminal and central regions have a degree of independence such that the two regions can bind simultaneously with two different lipid bilayers (Fusco et al., 2016b). This observation prompted the definition of a “double-anchor” mechanism, in which the N-terminal and the central regions of αS bind transiently across two different vesicles, thereby promoting their indirect interaction (Figure 1A). This mechanism, which is enhanced upon calcium binding at the C-terminal (Lautenschlager et al., 2018), was also shown to enable the stabilization of the docking of SVs onto the neuronal plasma membrane (Man et al., 2021).

In order to understand the mechanism of membrane interaction by the central region of αS in the context of the double-anchor mechanism (Fusco et al., 2016b), we here carried out an *in silico* investigation based on enhanced molecular dynamics (MD) simulations using coarse-grained (CG) force fields (Navarro-Paya et al., 2020). The data enabled clarifying the conformational dependency in membrane binding by the region 65–97 of αS and showed that the competition between tethered *versus* helical conformations has distinctive properties in this region, suggesting a role for vesicle recognition within the double-anchor mechanism. These results add to our understanding of the functional properties of αS in the context of synaptic vesicle binding.

## Materials and Methods

In order to characterize new mechanistic aspects of the double-anchor mechanism (Fusco et al., 2016b), we studied the modes of binding the region spanning residues 65–97 of αS (αS_65–97_) with DOPE:DOPS:DOPC lipid bilayers mimicking the lipid composition of SV, using CG MD simulations based on a modified version of the Martini 3 force field (Navarro-Paya et al., 2020). In this model, the motions of the backbone atoms are restrained to adopt two main conformational basins, respectively, accounting for extended-disordered and helical conformations.

The simulated system included one molecule of αS_65–97_, modeled with uncharged termini groups of the backbone, a lipid bilayer composed of 167 DOPE:DOPS:DOPC molecules per leaflet in a 84:50:33 (5:3:2) ratio, 8,728 martini water beads, and Na^+^ and Cl^−^ ions at a concentration of 150 mM. In the starting configuration of each trajectory, the center of mass of αS_65–97_ was positioned at a distance of 4.0 nm from the membrane surface. 15 independent simulations were run for 4.8 μs each at temperatures of 310–450 K (with a step increase of 10 K). These followed an equilibration phase performed at constant pressure until convergence of the area occupied by the lipids. The trajectories across the spectrum of temperatures were analyzed to generate melting curves of membrane binding and to analyze the conformational dependencies of membrane interaction by αS_65–97_.

### Simulation Setup

The GROMACS 4.6.7 package (Abraham et al., 2015) and a modified version (Navarro-Paya et al., 2020) of the Martini 3 force field (Bruininks et al., 2019) (see below) were employed in CG simulations of membrane-binding binding by the region 65–97 of αS (αS_65–97_). The composition of the synaptic-like membrane employed in this study recalls previous experimental (Fusco et al., 2016b) and *in silico* (Navarro-Paya et al., 2020) investigations and includes DOPE, DOPS, and DOPC lipid molecules mixed at a 5:3:2 (w/w) ratio (167 lipids in total). The protein was modeled with neutral termini, and the membrane component was generated using the Martini tool insane.py (Wassenaar et al., 2015). The peptide component was generated using the coarse-graining tool martinize.py starting from the full-atomistic structure of aS65-97 in helical or extended-disordered states. The system was solvated using Martini water models, and Cl− and Na + ions were added up to a concentration of 150 mM. For each simulation, the starting position included the protein with the center of mass positioned at a distance of 4 nm on the *z*-axis from the membrane component. The system was then equilibrated at different temperatures, with a series of 10 ns CG MD simulations in the NPT ensemble with 10 fs as the integration timestep interval. Thermal equilibration was run using the velocity-rescale thermostat (Bussi et al., 2007).

In each system, fifteen equilibration runs were performed at temperatures of 310–450 K (using a step interval of 10 K), with a thermal coupling constant of 2 ps and three distinct coupling groups (water molecules, ions, peptides, and lipids). Pressure was coupled at 1 bar using a semi-isotropic Berendsen barostat (Berendsen et al., 1984), with the *xy*- and *z*-axes coupled independently with a relaxation time of 12 ps and a 3 × 10^−4^ bar^−1^ compressibility. Subsequently, sampling runs were performed with 20 fs integration timestep for 4.8 μs each. The 15 samplings, which followed the temperature scheme of the equilibration runs (310–450 K), were thermalized using the velocity-rescale thermostat (Bussi et al., 2007), while pressure was coupled with the Parrinello–Rahman barostat (Parrinello and Rahman, 1981). Electrostatic interactions were accounted for with a coulomb cut-off of 1.1 nm, and van der Waals interactions were implemented with a cut-off of 1.1 nm. The Lincs algorithm (Hess et al., 1997) was used to constrain bond lengths and sidechain angles. The convergence of the simulations was assessed by dividing each run into three consecutive and equal segments and checking the convergence of the analysis of the membrane contacts.

### Modification of the Martini 3 Forcefield

As previously reported (Navarro-Paya et al., 2020), the Martini 3 forcefield was modified using angle restraints that restrain the backbone conformation to essentially two states, namely, α-helical and extended-disordered. These restraints act on the backbone atoms of the Martini 3 force field and include angles between three consecutive backbone particles $\left(\theta_{ijk}\right)$ and dihedral angles formed by four consecutive backbone particles $\left({\text{φ}}_{\text{ijkl}}\right)$ .

The angle $\left(\theta_{ijk}\right)$ is defined as

$${\text{θ}}_{\text{ijk}}=\cos^{-1}(\frac{\vec{ij}\cdot \vec{kj}}{\|\vec{ij}\|\|\vec{kj}\|}(,$$

and the restraining Gaussian is potential as

$${\text{V}}_{\text{θ}}=-{\text{K}}_{\text{ijk}}{\text{e}}^{-\frac{{\left({\text{θ}}_{\text{ijk}}-{\text{θ}}_{\text{min}}\right)}^{2}}{\text{σ}}}.$$

The resulting force applied on consecutive particles *i*, *j*, and *k*, is given by

$${\vec{F}}_{\theta }=-\frac{dV_{\theta }}{d\vec{r}}=-\frac{dV_{\theta }}{d\theta }\frac{d\theta }{d\vec{r}}=-\frac{2K_{ijk}\left(\theta_{ijk}-\;\theta_{min}\right)e^{-\frac{{\left(\theta_{ijk}-\theta_{min}\right)}^{2}}{\sigma }}}{\sigma }\frac{d\theta }{d\vec{r}}.$$

The angle $\left({\text{φ}}_{\text{ijkl}}\right)$ is defined from −180 to 180° using the four-quadrant inverse tangent as follows:

$$\cos\left({\text{φ}}_{\text{ijkl}}\right)=\frac{\left(\vec{ij}\times \vec{jk}\right)\cdot \left(\vec{jk}\times \vec{lk}\right)}{\left\|\vec{ij}\times \vec{jk}\right\|\left\|\vec{jk}\times \vec{lk}\right\|},$$

$$\sin\left({\text{φ}}_{\text{ijkl}}\right)=\frac{\vec{jk}\cdot \left[\left(\vec{ij}\times \vec{jk}\right)\times \left(\vec{jk}\times \vec{lk}\right)\right]}{\left\|\vec{jk}\right\|\;\left\|\vec{ij}\times \vec{jk}\right\|\;\left\|\vec{jk}\times \vec{lk}\right\|},$$

$${\text{φ}}_{\text{ijkl}}=\text{atan}2\left(\sin\left({\text{φ}}_{\text{ijkl}}\right),\cos\left({\text{φ}}_{\text{ijkl}}\right)\right),$$

and the restraining Gaussian potential is defined as

$${\text{V}}_{\text{φ}}=-{\text{K}}_{\text{ijkl}}{\text{e}}^{-\frac{{\left({\text{φ}}_{\text{ijkl}}-{\text{φ}}_{\text{min}}\right)}^{2}}{\text{σ}}}.$$

The resulting force acting on consecutive particles *i*, *j*, *k*, and *l* is given by

$${\vec{F}}_{\varphi }=-\frac{dV_{\varphi }}{d\vec{r}}=-\frac{dV_{\varphi }}{d\varphi }\frac{d\varphi }{d\vec{r}}=-\frac{2K_{ijkl}\left(\varphi_{ijkl}-\varphi_{min}\right)e^{-\frac{{\left(\varphi_{ijkl}-\;\varphi_{min}\right)}^{2}}{\sigma }}}{\sigma }\frac{d\varphi }{d\vec{r}}.$$

A detailed description of the individual parameters was reported previously (Navarro-Paya et al., 2020).

### Umbrella Sampling Full Atomic Simulations

Full atom simulations of the region 65–97 of αS (αS_65–97_) were carried out with the GROMACS 4.6.7 package (Abraham et al., 2015) using the amber99sb-ildn (Lindorff-Larsen et al., 2010) force field. Potential mean forces (PMF) were calculated with this method to estimate the membrane-binding free energy of αS_65–97_ in *a*-helix and extended-disordered conformations. In particular, umbrella sampling simulations were performed along a reaction coordinate defined as the distance between the centers of mass (COM) of the protein and the lipid bilayer. The latter was composed of a mixture of DOPE, DOPS, and DOPC lipids in a ratio of 5:3:2 as in the CG simulations. Initial conformations of αS_65–97_ in α-helix and extended-disordered conformations bound to the lipid bilayer were generated by extracting representative conformations from the Martini CG. These were backmapped into full atomic models of the protein and the membrane and solvated with tip3p waters. In order to obtain starting configurations of the umbrella sampling, αS_65–97_ was pulled away from the membrane along the normal direction to the lipid bilayer using a full atom MD simulation in the NPT ensemble. A total of 11 umbrella samplings were run to cover a path of 1.2 nm of αS_65–97_ from membrane-bound to membrane-detached conformation. The samplings consisted of 10 ns MD simulations, which followed 100 ps of equilibration, in the NPT ensemble, with an integration timestep interval of 2 fs at 300 K. Thermal equilibration was run using the velocity-rescale thermostat (Bussi et al., 2007). Pressure was coupled at 1 bar using a semi-isotropic Berendsen barostat (Berendsen et al., 1984), where the *xy* dimensions and the *z*-axis are coupled independently with a relaxation time of 1 ps and a 4.5 × 10^–5^ bar^−1^ compressibility. The COM distance of each window was restrained with a harmonic potential with a force constant of 1,000 kJ/mol. The PMF was calculated using the Weighted Histogram Analysis Method (WHAM) using the g_wham utility in GROMACS to calculate the ΔG of protein–membrane interaction along the pathway. The overlap of the individual umbrella simulations was used to assess the convergence of the sampling (Supplementary Figure S1).

## Results

**FIGURE 2:**
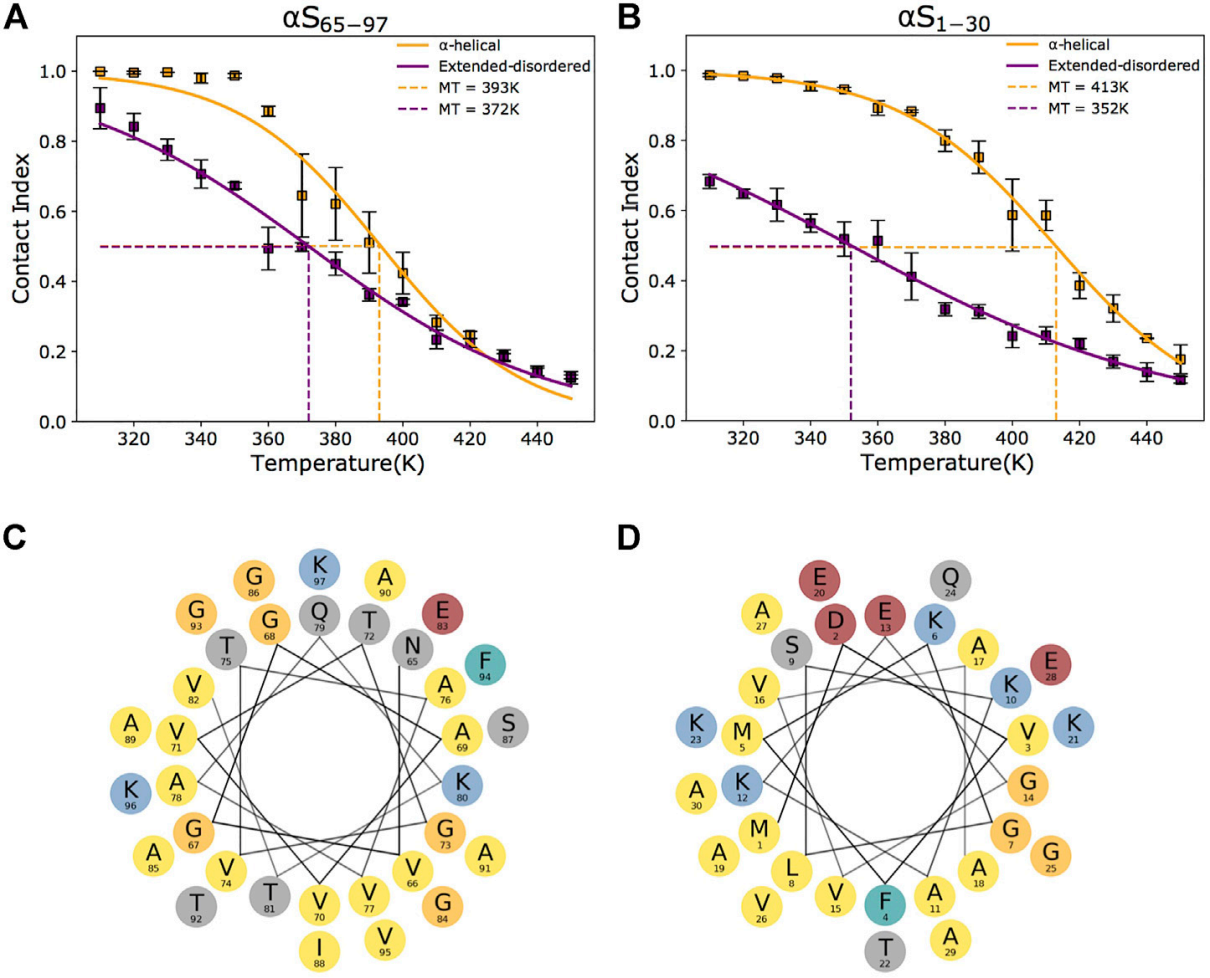
Conformational dependencies in membrane binding. **(A–B)** Melting curves of membrane binding based on the global contact index plotted as a function of the simulation temperatures. Purple and yellow lines report the melting curves calculated with the protein in helical and extended-disordered conformations, respectively. Binding curves to DOPE:DOPS:DOPC lipid bilayers by αS_65–97_ and αS_1–30_ are shown in panels **(A)** and **(B)**, respectively. Error bars report the standard deviation between three segments of the simulation. Data for αS_1–30_ are reproduced from Navarro-Paya et al. (2020). The difference in the melting temperatures in helical and extended-disordered conformations is attenuated in the case of αS_65–97_ compared to αS_65–97_. **(C–D)** Helical projections of the αS sequence reveal the amphipathic nature of αS_65–97_ **(C)** and αS_1–30_ **(D),** generating a hydrophobic surface (here pointing down in the plot) that is opposite to a hydrophilic (here pointing up in the plot). The amphipathic patterns are highly regular in the N-terminal region and residues and become imperfect in the region 65–97.

**FIGURE 3:**
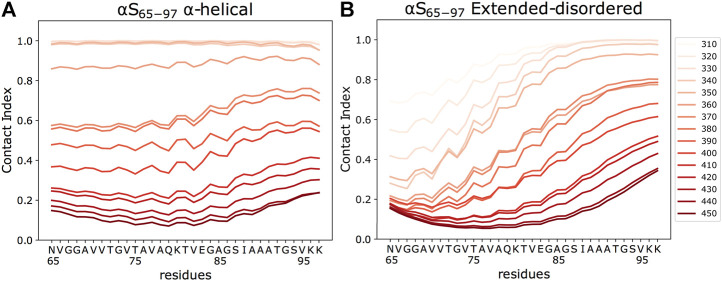
Residue-specific contact indexes. Plots are reported in temperatures ranging from 310 (light red) to 450 K (dark red), with a step increment of 10 K. Contact indexes for αS_65–97_ were computed for the binding to DOPE:DOPS:DOPC lipid bilayers in helical **(A)** and extended-disordered **(B)** conformations.

The overall goal of this study was to assess the conformational dependencies of membrane binding by the region 65–97 of αS (αS_65–97_) and to elucidate the modes of action of the second anchor in the double-anchor mechanism (Figure 1). The driving forces of the interaction between αS and synaptic membranes are complex and involve both steps of protein–membrane interaction and folding-upon-binding. In order to elucidate the conformational dependencies of the first term, we here used a computational framework based on a modification of the Martini force field for biomolecular simulations (Navarro-Paya et al., 2020) that restricts the conformational properties of the protein into extended disordered or helical states. The first state describes a “tethered” conformation of αS absorbed onto the membrane surface in an unstructured manner, whereas the second describes a “locked” membrane-bound state *via* an amphipathic protein conformation (Navarro-Paya et al., 2020). The analyses of the trajectories were based on the evaluation of the membrane-binding probabilities of each residue of the protein. This was computed using a contact index based on the minimum distance between Cα atoms of the protein and phosphate atoms of the lipids, with a threshold of 1 nm, and averaged across the whole trajectories (Figure 2). In addition, a global contact index, which is derived from the averaging of the contact indexes of the whole protein construct, was evaluated as a function of the temperature, providing melting curves of membrane interaction that directly account for the overall binding affinity of the protein (Figure 3). The melting curves from three independent segments of the trajectories were also employed to assess the convergence of the samplings (Supplementary Figure S2).

The analysis of the membrane interaction of αS_65–97_ indicates that the backbone conformations influence its membrane affinity. In particular, when binding in an extended-disordered conformation, the melting temperature of membrane binding was 372 K (Figure 2A). This value, which has a relative physical meaning and should be discussed within the framework of the CG simulations, increases when the simulations are performed with αS_65–97_ restrained in the helical conformation (393 K), indicating higher membrane affinity for the structured conformation. This finding is in line with full atomic simulations of umbrella sampling (Methods), indicating binding free energies of 11 and 7.5 kcal/mol for helical and extended-disordered conformations, respectively (Supplementary Figure S1). The observed difference of 21 K in the melting temperatures of the two conformations of αS_65–97_ in the CG simulations, however, is almost three times smaller than the difference associated with the construct of 1–30 residues (αS_1–30_, Figure 2B) (Navarro-Paya et al., 2020). The finding that for αS_65–97_, the binding to membranes in a disordered-tethered conformation is not as energetically disfavored as in the case of αS_1–30_ is ascribed to two concomitant factors. First, the membrane interaction in the helical-locked conformation is less stable in αS_65–97_ than in αS_1–30_ (melting temperatures of 392 and 413K, respectively), likely due to an imperfect amphipathic pattern of residues in αS_65–97_ with respect to αS_1–30_ (Figures 2C,D). Second, the membrane interaction by the extended-disordered state is stabilized in αS_65–97_ with respect to αS_1-30_ (melting temperatures of 372 and 352 K, respectively). Stabilization is particularly relevant for the region 87-SIAAATGSVKK-97, showing high membrane contact indexes throughout its sequence (Figure 3B) with values comparable to those of the helical conformation (Figure 3A). Indeed, for segments 87–97, we observed no difference between the melting curves calculated in the simulations of the extended-disordered and helical states (Supplementary Figure S3). This finding suggests that αS_87–97_ binds synaptic membranes in a conformational independent manner, with disordered-tethered *versus* helical-locked conformations having effectively the same membrane affinity.

## Discussion

Membrane binding is a crucial element for the pathophysiology of αS. This interaction plays a role in αS aggregation by influencing both the kinetics of self-assembly (Snead and Eliezer, 2014) and the mechanisms leading to the toxicity of αS oligomers (Fusco et al., 2017). The transient interaction with synaptic membranes is also a recursive element of most of the putative biological functions of αS, such as those involved in the regulation of the homeostasis of synaptic vesicles (SVs) (Gitler et al., 2008; Soper et al., 2008; Auluck et al., 2010; Burre et al., 2010; Diao et al., 2013; Burre et al., 2014). αS was indeed detected as a “SV visitor” protein in ultra-definition proteomic studies (Taoufiq et al., 2019), confirming the transient nature of its interaction with SVs as probed with NMR (Fusco et al., 2014), and was found to colocalize with SVs in synaptosomes in a calcium-dependent manner (Lautenschlager et al., 2018). Upon membrane binding, αS adopts conformations that endow it with the ability to mediate membrane–membrane interactions (Fusco et al., 2016b), such as those responsible for the mediation of vesicular clustering as observed *in vitro* using model vesicles (Bodner et al., 2009; Diao et al., 2013; Man et al., 2020) and *ex vivo* SVs (Fusco et al., 2016b), as well as in the cellular environment (Gitler et al., 2008; Soper et al., 2008). In the context of SV homeostasis, SV clustering by αS has been associated with the maintenance of distal pools of SVs at the presynaptic membrane, thereby helping to regulate the number of synaptic vesicles docked at the synapse (Cooper et al., 2006; Wislet-Gendebien et al., 2006; Auluck et al., 2010).

NMR investigations revealed an underlying double-anchor mechanism at the origin of the mediation of membrane-membrane interactions and vesicular clustering by αS (Fusco et al., 2016b). The mechanism involves the binding of one vesicle *via* the N-terminal region of the protein (first anchor) and a second vesicle *via* the region 65–97 (second anchor, Figure 1). The membrane interaction of the first anchor has been characterized extensively by means of NMR experiments (Fusco et al., 2016a; Runfola et al., 2020) and biomolecular simulations (Fusco et al., 2016a), showing that the helical-locked conformation is essential for the stabilization of the bound state (Navarro-Paya et al., 2020), with the initial 12 residues inserting into the hydrophobic part of the lipid bilayer (Fusco et al., 2016a). It is, however, unclear what the mechanism of membrane interaction of the second anchor (residues 65–97) is.

The present study exploited a computational framework of enhanced CG MD simulations to investigate the conformational dependencies in membrane binding by αS_65–97_. The data indicated that, for this region, the interaction with the membrane in the disordered-tethered conformation is energetically more favorable than for the N-terminal membrane anchor of the protein. This feature, combined with a non-optimal amphipathic profile in the helical conformation of the region 65–97 (Figures 2C,D), minimizes the difference between the membrane affinities of the disordered-tethered and helical-locked conformations, compared to the N-terminal case. In the extreme case of segments 87–97, the membrane-binding curves of these two conformations were found to completely converge (Supplementary Figure S2), indicating that locally αS binds synaptic membranes in a conformation-independent manner. This finding is in agreement with the experimental observation that the deletion of residues 36–42 and 45–57 reduces the overall helical content of αS upon binding with DMPS vesicles, as observed *via* circular dichroism (CD), while simultaneously increasing the local membrane interaction of the region 65–97, as observed *via* the broadening of the NMR ^1^H-^15^N-HSQC resonances, thus indicating a binding with low helical content for this region in the truncated mutants (Doherty et al., 2020).

Our results may have key implications for the double-anchor mechanism. Previous studies showed that the exposure of the region 65–97 in the membrane-bound state of αS is a dominant factor in promoting vesicle–vesicle interactions (Fusco et al., 2016b). From these studies, it was found that the active conformation of αS for the double-anchor mechanism includes the membrane anchoring through an N-terminal helix and the protrusion of the region 65–97 away from the membrane surface, with the latter acting as an antenna to sense a second vesicle (state B* in Supplementary Figure S4). As this active conformation of membrane-bound αS is only transiently populated in the conformational ensemble (Man et al., 2020), its ability to engage in interactions with a second vesicle also relies on kinetic factors. In this context, the present finding that, in the region 87–97, membrane binding is independent of the protein conformation provides evidence of a kinetically simplified binding mechanism that does not require a step of folding-upon-binding. We postulate that the identified modes of binding might have functional implications for the second anchor in the double-anchor mechanism to facilitate vesicle clustering by αS.

In conclusion, this study adds to the understanding of the membrane interaction of αS in view of the functional binding of the region 65–97 and its role in SV clustering *via* the double-anchor mechanism. This region is also relevant for the pathological aggregation into toxic oligomers (Fusco et al., 2017) and largely overlaps with the amyloidogenic NAC segment of αS (Ueda et al., 1993; Uversky and Eliezer, 2009). In addition to exhibiting plasticity in adopting different conformations, that is, from the random coil in the cytosol (Theillet et al., 2016) to α-helix in the membrane bound (Bodner et al., 2009) and to β-sheet in the amyloid (Tuttle et al., 2016; Li et al., 2018), the central region of αS has been shown to have a promiscuous tendency to bind membranes both in disordered-tethered or helical-locked conformations. The chameleon nature of this region, which also hosts a regulation site *via* the phosphorylation of residues Ser 87 (Oueslati et al., 2012), is likely to be at the origin of the multiplicity of putative functions of αS but may also have aberrant roles in promoting unwanted interactions under conditions leading to αS aggregation in PD.

## Data Availability

The original contributions presented in the study are included in the article/Supplementary Material. Further inquiries can be directed to the corresponding author.
