# Supplementary material for: Plasticity of Membrane Binding by the Central Region of α-Synuclein
Source: Front Mol Biosci. 2022 Jun 15;9:857217. doi: 10.3389/fmolb.2022.857217 (PMC9240306; doi:10.3389/fmolb.2022.857217)
Supplement: Supplementary file 1 [file DataSheet1.pdf]

Supplementary Material

Plasticity of membrane binding by the central region of α-synuclein

Carlos Navarro-Paya[1], Maximo Sanz-Hernandez[1], Alfonso De Simone[2,1] *

[1]Department of Life Sciences, Imperial College London, South Kensington, SW7 2AX, UK

[2]Department of Pharmacy, University of Naples "Federico II", via Montesano 49 Naples, 80131, Italy

*** Correspondence:**
Alfonso De Simone
alfonso.desimone@unina.it

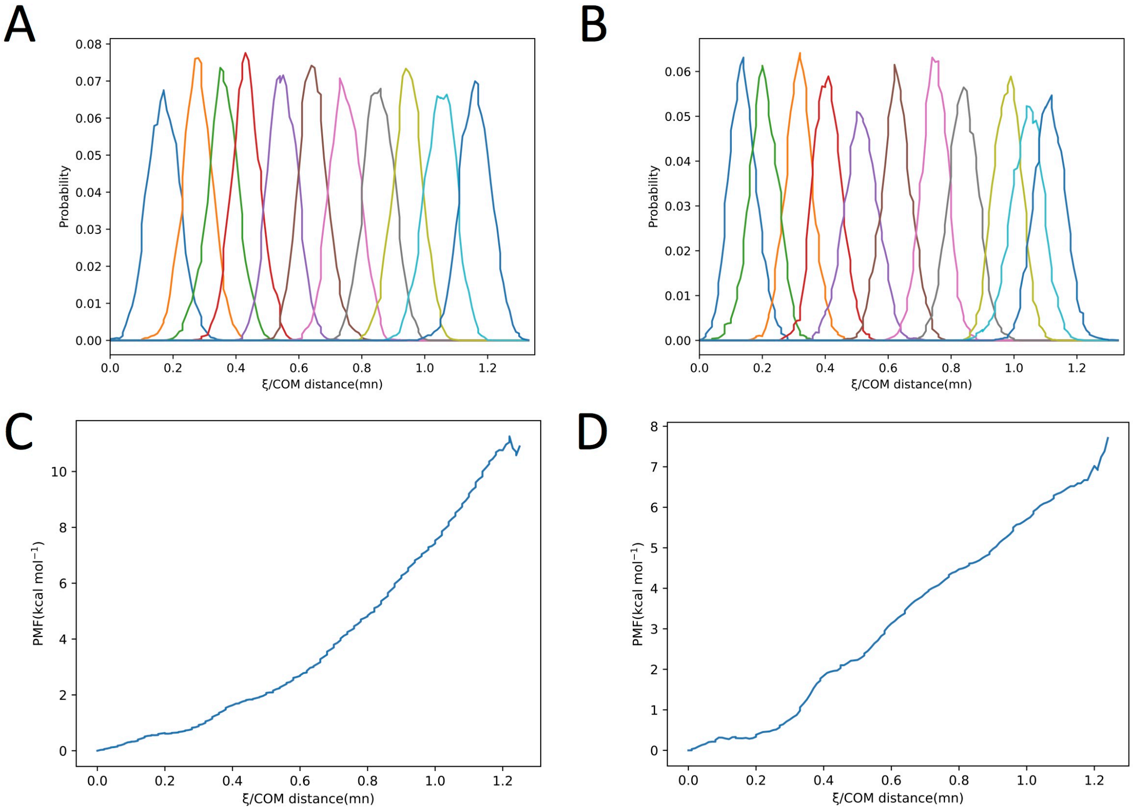

Figure S1. Full atom umbrella sampling of α$S_{65–97}$ membrane binding. A-B) Convergence of the umbrella sampling assessed from the overlap of the distribution of distances of the COM in the independent 13 simulations of each sampling. Plots for αS_{65-97} binding to DOPE:DOPS:DOPC lipid bilayers in the helical (A) and extended-disordered (B) conformations are shown. C-D) Potential mean force of binding of αS_{65-97} to DOPE:DOPS:DOPC lipid bilayers in the helical (C) and extended-disordered (D) conformations. The free energy profile was obtained with the WHAM method by analyzing the histograms of distributions in the umbrella samplings.

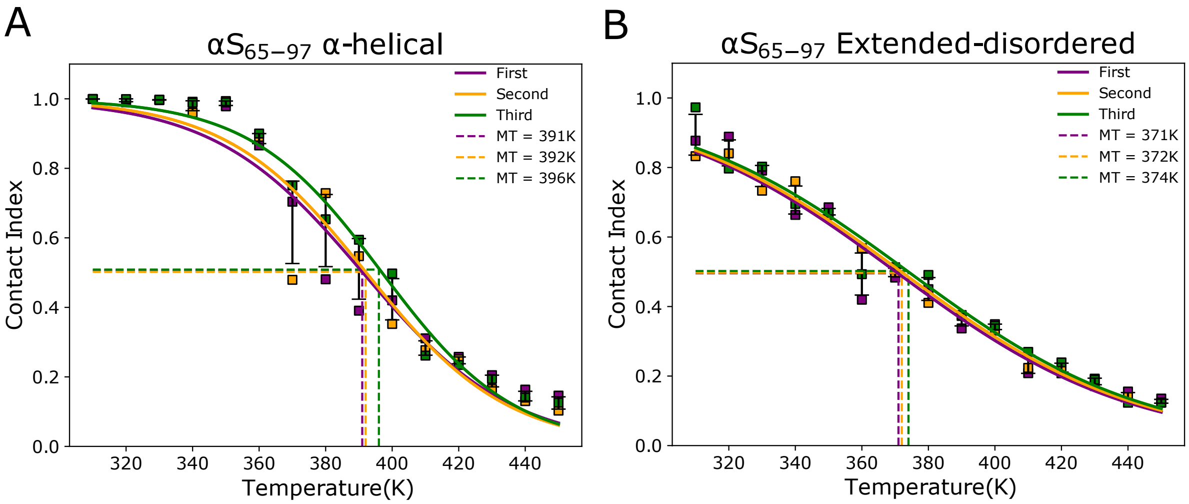

Figure S2. Convergence simulations of αS$_{65–97}$ membrane binding. Convergence was assessed by dividing the trajectories into three equivalent and consecutive segments and by comparing observables calculated in these sub-samplings. Membrane-binding melting curves were calculated from plotting the global contact index as a function of the temperature of the simulation. Plots for αS$_{65-97}$ binding to DOPE:DOPS:DOPC lipid bilayers in the helical (A) and extended-disordered (B) conformations are shown. First, second and third segments of the simulations are shown in purple, orange and green, respectively.

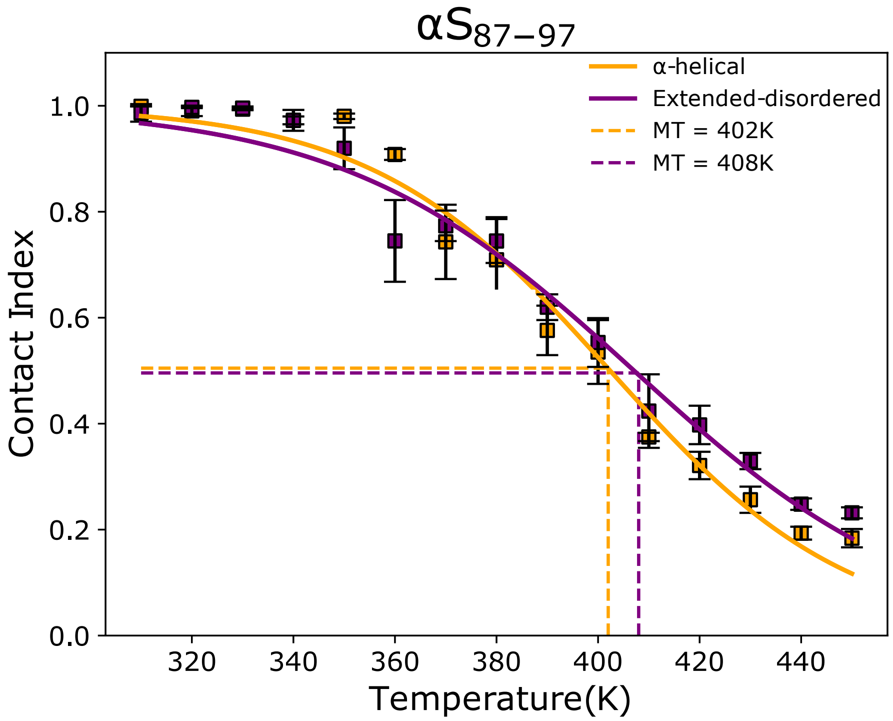

Figure S3. Melting curves of membrane-binding in the region αS65-97. Plots report the global contact index as a function of the simulation temperatures. Purple and yellow lines report the melting curves calculated with the protein in helical and extended-disordered conformations, respectively. Error bars report the standard deviation between three segments of the simulation. The plot shows no difference in the melting temperatures in helical and extended-disordered conformations for the αS87-97 region.

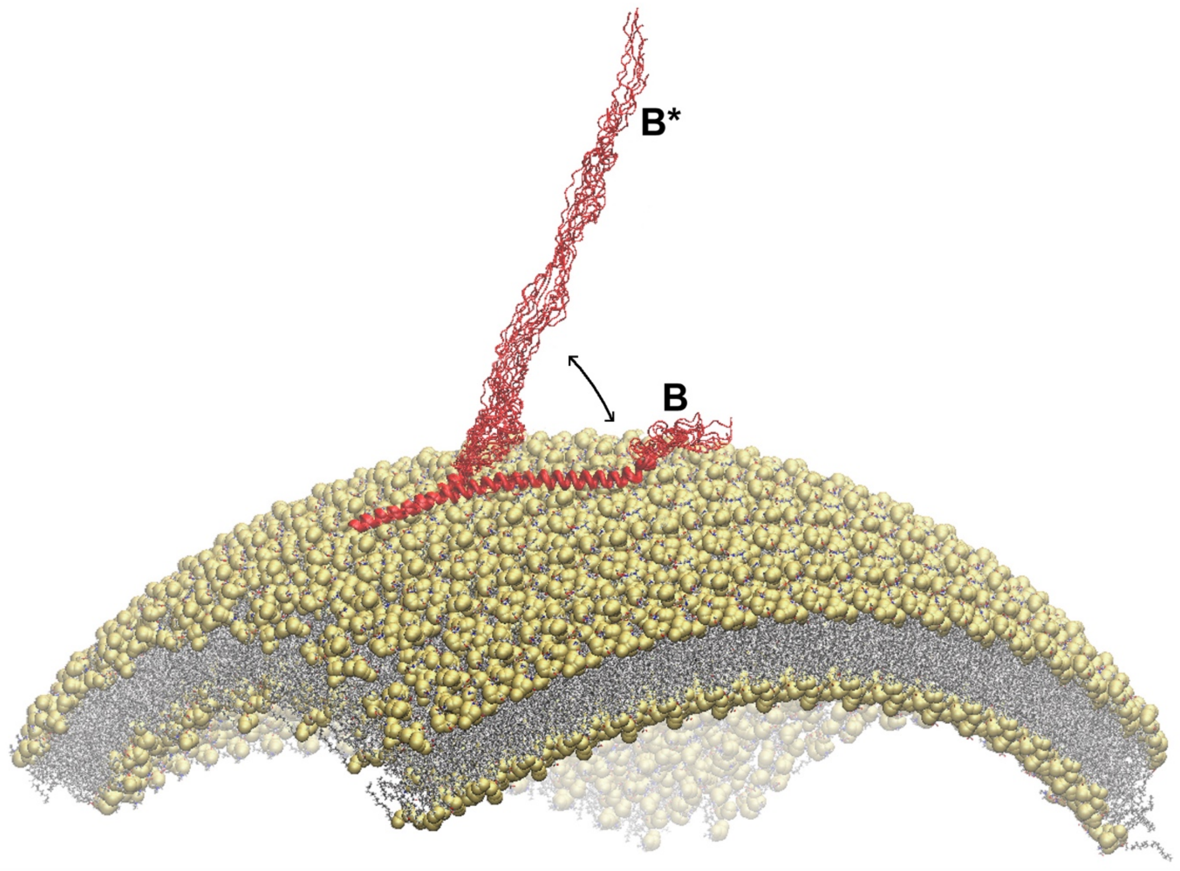

Figure S4. Schematic representation of the conformational exchange in the membrane-bound state of αS. The bound state is shown to adopt two major conformational basins, namely a fully α-helical state spanning the whole region 1-97 bound to the membrane (state B) and a state B* where only the N-terminal region (residues 1-25) is bound to the lipid bilayer, with the rest of the protein being detached from the membrane surface. The state B* is an active conformation for the recruitment of a second vesicle through the double-anchor mechanism (Fusco et al., 2016b).
